# Genetic diversity of Bm86 sequences in *Rhipicephalus (Boophilus) microplus* ticks from Mexico: analysis of haplotype distribution patterns

**DOI:** 10.1186/s12863-019-0754-8

**Published:** 2019-07-12

**Authors:** S. G. Martínez-Arzate, J. C. Sánchez-Bermúdez, S. Sotelo-Gómez, H. M. Diaz-Albiter, W. Hegazy-Hassan, E. Tenorio-Borroto, A. Barbabosa-Pliego, J. C. Vázquez-Chagoyán

**Affiliations:** 1 Centro de Investigación y Estudios Avanzados en Salud Animal, Facultad de Medicina Veterinaria y Zootecnia, Universidad Autónoma del Estado de México, Kilometro 15.5 Carretera Panamericana, CP 50200 Toluca-Atlacomulco, Mexico; 2 Wellcome Centre for Molecular Parasitology, University of Glasgow, University Place, Glasgow, G12 8TA UK; 3 Colegio de la Frontera del Sur, Carretera Villahermosa-Reforma Km 15.5, Ranchería Guineo, sección II, CP 86280 Villahermosa, Tabasco Mexico

**Keywords:** Tick, *R. microplus*, Bm86, Diversity, Haplotype

## Abstract

**Background:**

Ticks are a problem for cattle production mainly in tropical and subtropical regions, because they generate great economic losses. Acaricides and vaccines have been used to try to keep tick populations under control. This has been proven difficult given the resistance to acaricides and vaccines observed in ticks. Resistance to protein rBm86-based vaccines has been associated with the genetic diversity of Bm86 among the ectoparasite’s populations. So far, neither genetic diversity, nor spatial distribution of circulating Bm86 haplotypes, have been studied within the Mexican territory. Here, we explored the genetic diversity of 125 Bm86 cDNA gene sequences from *R. microplus* from 10 endemic areas of Mexico by analyzing haplotype distribution patterns to help in understanding the population genetic structure of Mexican ticks.

**Results:**

Our results showed an average nucleotide identity among the Mexican isolates of 98.3%, ranging from 91.1 to 100%. Divergence between the Mexican and Yeerongpilly (the Bm86 reference vaccine antigen) sequences ranged from 3.1 to 7.4%. Based on the geographic distribution of Bm86 haplotypes in Mexico, our results suggest gene flow occurrence within different regions of the Mexican territory, and even the USA.

**Conclusions:**

The polymorphism of Bm86 found in the populations included in this study, could account for the poor efficacy of the current Bm86 antigen based commercial vaccine in many regions of Mexico. Our data may contribute towards designing new, highly-specific, Bm86 antigen vaccine candidates against *R. microplus* circulating in Mexico.

## Background

Ticks are obligated hematophagous ectoparasites that feed on a wide variety of mammals, including humans and cattle. *Rhipicephalus microplus* (Acari: Ixodidae) is a cattle one-host-tick, with a tropical and subtropical cosmopolitan distribution. It is considered one of the most important ectoparasites of cattle in Mexico, negatively affecting weight gain and milk production which results in substantial economic losses. Additionally, this species is able to transmit dangerous diseases to cattle such as babesiosis and anaplasmosis [1, 2].

In Mexico, eradication campaigns against *R. microplus* have been conducted for over 60 years [3]. Campaigns included strategies aiming to keep geographic regions free of ticks, as well as actions towards control and eradication of the ectoparasite from endemic areas (Mexican Official Standard NOM-019-ZOO-1994). Such actions are achieved mostly through application of acaricides which are currently presenting limited efficacy, as ticks have developed resistance, an issue that has been widely discussed in previous researches [4–9].

This is an escalating problem in Mexico for cattle production, due to the presence of various drug-resistant and multi-resistant ticks [10–17]. Furthermore, the continuous use of chemicals in tick control results in environmental pollution, as well as contamination of dairy and meat products [18–20].

Given the variability of resistance mechanisms to acaricides presented by ticks, alternative control methods have been explored. These include pheromone decoys for attracting and killing ticks, selection of cattle with natural resistance to ticks, biological control agents, as well as the administration of anti-tick vaccines [21, 22]. The successful use of vaccines to combat ticks was first published by Allen and Humphreys using tick midgut extracts [23]. Bm86 is a tick midgut protein, which has been used since the 1990s as a vaccine antigen [24–26]. Three vaccines with protection efficacy against *R. microplus* have been commercialized since then: Gavac® (Heber Biotec, Havana, Cuba), TickGARD® (Hoechst, Animal Health; Australia), and TickGARD PLUS® (Intervet, Australia), [22, 24, 26–30].

The rBm86 protein-based vaccine, is marketed in several countries of Latin America, Asia and Oceania, showing a variable efficacy, for example; the combined use of the anti-tick vaccine and strategic acaricide treatments, showed a reduction in the consumption of acaricides of 87% [27] and 95% [31] in Cuba and 90% in Australia [28], while in Mexico the reduction in use of acaricides could be only reduced by 67% [27]. The effectiveness of the vaccine has been associated with amino acid variations of the protein among different populations of *R. microplus* [19, 22, 27, 32–34]. This was observed in a particular variant of the Bm86 antigen in Argentina. The antigen was renamed as Bm95, and it is currently used as an alternative vaccine antigen [31, 35].

Genetic polymorphism has resulted in diverse Bm86 haplotypes in different geographical regions of the world [26, 35–38], so a vaccine should be designed considering regional antigenic variants. Exploring the haplotypes and haplogroups of the Bm86 sequences and their distribution within the phylogeographic structure, could help in designing better vaccines against *R. microplus*. In this study, phylogeographic aspects such as the spatial distribution of haplotypes and the genetic variability of a cDNA transcript sequence for the Bm86 gene from *R. microplus* collected from 10 Mexican states was assessed.

## Methods

### Experimental methods

#### Sampling

**Fig. 1:**
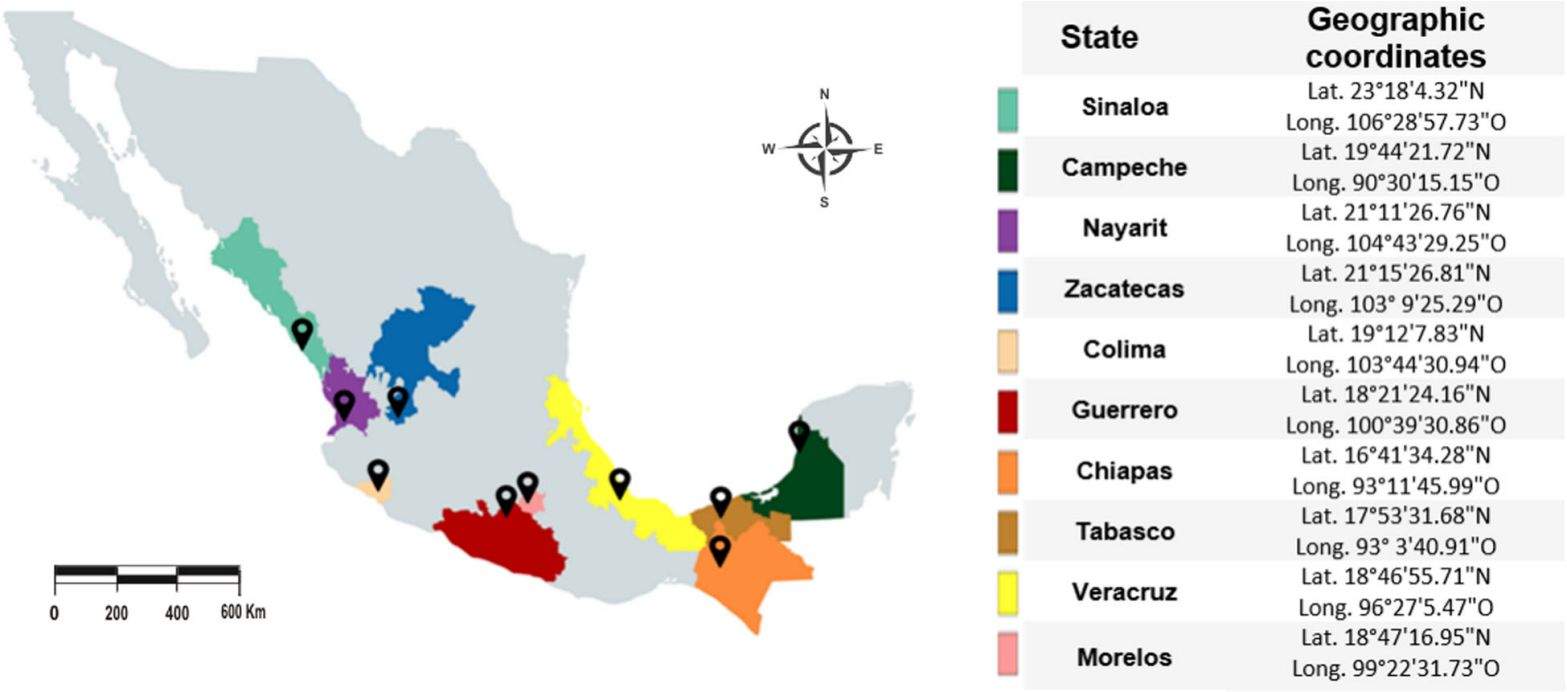
Collection sites map. Samples from *R. microplus* specimens from 10 states of Mexico were collected. Pacific, gulf, center and north center regions of the country were included. Each color corresponds to different collection state in Mexico and each black pin represents the approximate geographical coordinates of the location in the map for each collection site

Sampling protocol was approved by the Animal Care Committee and Bioethics (COByBA) of the Facultad de Medicina Veterinaria y Zootecnia of the Universidad Autónoma del Estado de México. Adult female ticks (*N* = 200, 20 / locality) were collected from February 2012 to July 2015, from 10 different localities belonging to 10 different states of the Mexican Republic (Campeche, Veracruz, Morelos, Colima, Sinaloa, Guerrero, Nayarit, Chiapas, Tabasco and Zacatecas; Fig. 1). All sampled localities are under the official tick control program, according to the National Campaign for *Boophilus* spp. Tick Control [39]. Ticks were collected directly from the skin of the cattle, with verbal consent of the animals’ owners. Individual animals, infested with ticks, were immobilized by the owners, and ticks were collected with entomological forceps and placed in 50 ml conical falcon tubes, adapted for tick transport with a lid with air filter, and a piece of folded paper and a humidifier within the tube. Tubes were kept within a styrofoam box to maintain temperature and humidity until arrival to the laboratory. Upon arrival, ticks were kept in an incubator at 30 °C and a relative humidity of 80–90% for no longer than 3 days, until dissected for the collection of the intestines. The species identification of collected specimens was based on the external morphology of *Rhipicephalus (Boophilus) microplus* according to Walker et al. [40].

#### RNA extraction

All dead arthropods (*n* = 25) were discarded, and total RNA from the remaining 175 ticks was individually extracted and purified from midguts using TRI Reagent® (Ambion, USA), according to the manufacturer’s instructions, as follows: 1) Homogenization: tissue (10–15 mg midgut) from individual ticks was placed in 1 ml of TRI Reagent solution, homogenized by vortex, followed by centrifugation at 12,000 xg, at 4 °C for 10 min. Supernatant was collected in a fresh microcentrifuge tube and stored at − 80 °C until further use, or used immediately, 2) RNA extraction: 100 μl of 1-bromo-3-chloropropane per 1 mL of TRI Reagent solution were added, followed by centrifugation 12,000 xg, at 4 °C for 15 min and the aqueous phase was transferred to a fresh tube, 3) RNA precipitation: RNA was precipitated and resuspended by adding 500 μl of isopropanol followed by centrifugation at 12,000 xg, at 4 °C for 8 min, the supernatant was discarded, the RNA pellet washed with 1 mL of 75% ethanol, followed by centrifugation 7500×g, at 4 °C for 5 min, the ethanol was discarded and the pellet left to air dry 2–3 min, 4) RNA solubilization: the pellet, was resuspended in 40 μl of nuclease free water and stored at − 80 °C until further use [19, 39, 40]. The RNA obtained was quantified with a microvolume spectrophotometer (Quawell Q5000, USA) at 260 nm absorbance. RNA purity was estimated considering the absorbance readings at 230: 260: 280 OD, with ratios close to 1:2:1 were considered satisftory. The integrity of the RNA was verified and documented by agarose gel electrophoresis (1% agarose stained with ethidium bromide, run at 10 V / cm for 15 min).

#### cDNA synthesis

RNA from 170 ticks was successfully isolated and purified and was used to synthesize complementary DNA (cDNA), SuperScript II Reverse Transcriptase (Invitrogen, USA) was used according to the manufacturer’s instructions. The reaction mixture for a total volume of 12 μL was: 8 μL of nuclease-free water, 1 μL of Oligo (dT) 500 μg / ml, 2 μL of total sample RNA (at 2000 ng / μl), 1 μL of dNTPs (10 mM). The mixture was incubated in a thermocycler (Techne TC-512 USA) at 65 °C for 5 min and then 4 °C for 2 min. Then, 4 μL of 5 X First-Strand Buffer and 2 μL of DTT were added and incubated at 42 °C for 2 min. Finally, 1 μL reverse trascriptase SuperScript II was added, followed by a 42 °C incubation for 50 min [41–43]. RNA samples were subjected to a DNA digestion according to the DNA-free™ Kit protocol (Ambion), subsequently sample concentrations and purity evaluated using a microvolume spectrophotometer (Quawell Q5000) at Absorbance ratios A_260/A280_ and A_260/A230_. From 170 initial RNA samples, only 163 cDNA sequences were successfully produced.

#### Amplification of the target sequences and sequencing

To obtain the Bm86 cDNA target sequence, four pairs of primers (see Table 1) were designed using the Primer3 software (http://www.bioinformatics.nl/cgi-bin/primer3plus/primer3plus.cgi). Primers were designed based on the sequence of *R. microplus* reference strain Yeerongpilly, (NCBI access number M29321.1) as a template.

**Table 1:** Primers sequences used to amplify the Bm86 cDNA by rt-PCR

Primer		^a^Sequence	^b^Tm/sec	^c^Size (bp)
Bm86_	F1	5’-GTGGTTCGACGCAGTGAGAT-3′	60°/30	680
	R1	5’-CCATGCTTGCAGACAAACCC-3´		
	F2	5’-CTGCAAAGACCTCTGCGAGA-3´	55°/30	686
	R2	5’-TGTCCTGCGTGCAGTTAAGT-3´		
	F3	5’-CGGGCCCAAATGTCAACATC-3´	55°/30	680
	R3	5’-AACGCACTCCAGCTTCTTGT-3´		
	F4	5’-AAAACGAGCAGTCGGAGTGT-3´	57°/30	542
	R4	5’-GGTGTTCGATGTAAGCGTGA-3´		

^a^Primer sequence, directionality is indicated as forward (F) and reverse (R)

^b^Melting temperature in Celsius degrees/incubation time in seconds

^c^Amplicon size in base pairs are listed for each pair of primers

The primers were designed to produce amplicons with 200 bp overlapping flanking regions to cover the entire 1950 bp cDNA sequence. A total of 652 PCR reactions were prepared (163 × 4: four reactions per cDNA sample). The PCR thermal cycling reactions were performed in an automated thermal cycler (Arktik, 96-well gradient; Thermo Scientific) as follows: an initial step of denaturation at 94 °C for 4 min, followed by 30 cycles of denaturation at 94 °C for 1 min, alignment temperature for 30 s and extension at 72 °C for 1 min, and a final extension incubation at 72 °C for 5 min (primer sequences, alignment temperatures and amplicon sizes are shown in Table 1). Bm86 amplicon fragments were visually evaluated for size and integrity in a 1.5% agarose gel electrophoresis (30 min at 100 V) and stained with Ethidium Bromide [19, 41]. Negative (no DNA target sequences) and positive (a known cDNA fragment, containing the Yeerongpilly Bm86 gene) control reactions were included as PCR in every assay, to rule out contamination and to confirm that the reaction was set correctly.

From 652 PCR reactions, 600 amplicons were successfully recovered and products purified using a Qiaquick PCR Purification Kit (Qiagen, Netherlands). Amplicon concentrations were estimated with a microvolume spectrophotometer (Quawell Q5000) at OD_260_. Purity of amplicons were estimated using OD_230 / 260 / 280_ ratios. Six hundred samples containing 50 ng of cDNA in 10 μL volume, were individually packed and labeled in PCR tubes. Four overlapping amplicons per tick, representing the full putative Bm86 cDNA sequence of 150 tick samples, were sent for forward and reverse sequencing through the Sanger method to Genscript Corporation, Piscatway, NJ, USA (https://www.genscript.com).

### Bioinformatics methods

#### In silico sequence analysis

To verify sequences reliability, fasta DNA sequences were initially analyzed and assembled with the CodonCode Aligner sofware (CodonCode Corporation, www.codoncode.com). The primers amplified arround 600 bp (542–686 bp, Table1), with an overlap of approximately 200 bp with contiguous sequences. These overlaps allowed to optimize the full sequence assembly, and to confirm quality of overlapping sequences. Additionally, this strategy leaves a high resolution, optimal central reading sequence, of approximately 200 bp. All sequences were analyzed for completeness and high quality with the following parameters: 1) extremes with low resolution (not clearly defined chromatogram peaks) were eliminated from the sequences, 2) where double peaks were observed for a single position in the chromatogram, only the tallest peak was considered, 3) amplicon sequences that did not have large overlapping fragments (> 100 bp) with the contiguous Bm86 cDNA sequence region, were not considered for any further analysis. From 150 Bm86 cDNA tick samples sequenced by Genscript (corresponding to 600 amplicons sequenced), only 125 could be successfully assembled for the complete putative Bm86 cDNA sequence. All 125 sequences were included in a data matrix in Mega6 software [44] along with all the cDNA Bm86 sequences available in GenBank at the time this study was conducted. (https://blast.ncbi.nlm.nih.gov/Blast.cgi) [44–46]. Subsequently, a multiple alignment of all these sequences was performed with Mega 6 software using the global ClustalW method. This data matrix was used for different analyses.

#### Analysis of genetic variability

Genetic variability of 125 putative Bm86 cDNA gene sequences was analyzed in *R. microplus* collected from 10 different localities in Mexico (Table2). The trend of the haplotypes in the population was analyzed through nucleotide (the probability that two randomly-chosen homologous sites are different), and haplotype (the probability that two randomly-chosen haplotypes are different) diversity. The number of haplotypes and the values of nucleotide and haplotype diversity were calculated for each of the collection sites with the DNA sequence polymorphism software DnaSP 5.10. (http://www.ub.edu/dnasp/DnaSP_OS.html) [47].

#### Haplotype frequency

A 616 amino acid sequence, including residues 19–634 encoding for the mature Bm86 protein, was included in the analysis. All extra sequences obtained from the sequencing and analysis process were trimmed out. For the haplotype frequency analysis, nucleotide and haplotype diversity values were calculated with the DnaSP software, using the default parameter: Invariable sites were removed, sites with gaps/missing were not considered and the analysis generated was a NEXUS haplotype data file.

In order to evaluate the haplotypes frequency, we first specified the haplogroups (groups of sequences that share 100% identity in their amino acid sequence) and the frequency in which a determined haplotype appears in each haplogroup. Then, we determined the genetic constitution, as the proportion of sequences that belong to a haplogroup. Additionally, we calculated the degree of divergence of each haplotype, with respect to the reference sequence (Yeerongpilly). This degree of divergence corresponds to the sum of single nucleotide polymorphisms (SNPs), when comparing two aligned sequences, expressed as a percentage [48, 49].

#### Mantel test

A Mantel test was performed to explore whether a significant relationship existed between genetic and geographic distances among the sampled locations. The postulate is an island-like model, in which each population diverges at the same time. This was carried out to evaluate the haplotypes’ spatial structure, specifically if the observed haplotypes were confined to a geographic region in Mexico.

To generate the genetic distances matrix, an average value of the identity percentage was used for all possible population pairs. These values were calculated with MatGAT v2.01 (Matrix Global Alignment Tool) software [50] (http://www.angelfire.com/nj2/arabidopsis/MatGAT.html).

The matrix of geographic distances was constructed considering the geographic coordinates from each locality sampled and data fidelity was confirmed using Google Earth software (https://www.google.com/earth/download/ge/). The matrix represents the distance in km between each pair of tick populations analyzed. The data matrices were analyzed using the statistical software XLSTAT (https://www.xlstat.com/es/). The Pearson correlation function was used, which determines if there is a linear relationship between two variables. The correlation coefficients between the elements of each matrix were obtained using 10,000 permutations and a significance of 95%. The hypotheses considered were: H_1_ There is an isolation by distance for the sequence Bm86 in the different sampled populations; H_0_ There is not an isolation by distance for sequence Bm86 in the different sampled populations.

#### Phylogenetic analysis

Mega 6 software [44] was used to estimate hypothetical evolutionary distances among Bm86 putative genetic sequences of *Rhipicephalus microplus,* using 125 complete cDNA sequences from tick samples collected from 10 different regions from Mexico and 38 sequences published in GenBank. To evaluate clustering probabilities during tree construction, Maximum Likelihood statistical method and Kimura 2-parameter substitution model were used. The bootstrap confidence values were estimated using 1000 permutations. Discrete Gamma distribution was used to model the evolutionary rate differences among sites. The following complete Bm86 sequences from different regions of the world, retrieved from GenBank were included in the analysis: Australia (M29321), Argentina (AF150891), India (HQ166286), Mozambique (FJ809946, EU191620); Brazil, (EU352677), 14 from the USA (HQ014385 to HQ014398) and 18 from Thailand (KJ995882 to KJ9958910). As external sequences, two *R. annulatus* Bm86 homologous sequences, (HQ014399 and HQ014401) were included, [33].

A minimum spanning tree (MST) or minimum Expansion Network, was built using the Network publisher software (http://www.fluxus-engineering.com) [51]. For this analysis, we used the total number of haplotypes observed along with the global reports retrieved from GenBank. The coalescence and the connections between the haplotypes, given by the minimum number of mutations among them, were the basis of the analysis to generate a tree that shows the frequency and the relation with the distances in number of mutations that separate each haplotype from others. The Median Joining (MJ) method was chosen for the construction of the network, this method combines characteristics of the Kruskal and Farris algorithms. The first favors the short connections in the network for finding minimum spanning trees, while the second is a heuristic algorithm of maximum parsimony (MP) that adds the so-called median vectors.

## Results

### Genetic variability

In the present study, 125 full-length cDNA strands, encoding Bm86 mature protein code from 10 different localities in Mexico were analyzed. We found 145 single-nucleotide polymorphisms. Total haplotypic diversity was estimated in *h* = 0.017 and total nucleotide diversity in π = 0.012**.** Diversity values were calculated for each sampled locality. Chiapas was the locality with the highest haplotypic diversity (*h* = 1) from the 10 states sampled, while Zacatecas and Nayarit had the lowest value (*h* = 0.84). Regarding nucleotide diversity, the lowest value was observed in the locality of Veracruz (*π* = 0.001) and the highest value (*π* = 0.018) was observed for Zacatecas (Table 2).

**Table 2:** Genetic variability of the Bm86 gene in the different sampled localities

Locality	*n*	*n*_*h*_	*n*_*hu*_	*h*	*π*
Campeche	18	11	7	0.94	0.007
Veracruz	18	8	4	0.90	0.001
Morelos	13	12	11	0.98	0.013
Colima	14	9	7	0.89	0.014
Sinaloa	11	10	9	0.98	0.017
Guerrero	11	9	8	0.97	0.015
Nayarit	10	6	4	0.84	0.003
Chiapas	9	9	9	1	0.015
Tabasco	11	9	7	0.96	0.005
Zacatecas	10	6	4	0.84	0.018
*n* total	125				

*n* number of sequences analyzed

*n*_*h*_ Number of observed haplotypes

*n*_*hu*_ Number of unique haplotypes

*h* Haplotype diversity

*π* Nucleotide diversity

Note: Since some haplotypes from different localities are identical, the sum of observed haplotypes and sum of unique haplotypes of all localities does not correspond to the final number of haplotypes found in the study. To find this numbers please refer to Table 3

Sixty-four different haplotypes were identified from the genetic variance of the sequence Bm86. Nine of them formed haplogroups (see Table 3) or groups of sequences that shared a 100% identity within each group, the remaining 55 sequences, were unique haplotypes. Haplogroups 1 and 2 represent, respectively, 22.2 and 14.2%, of the total number of sequences analyzed, both were observed in 9 of the 10 sampled localities. The percentage of divergence for these 9 haplogroups remained between 3.1–7.4% (Table 3**).**

**Table 3:** Haplogroups for sequence Bm86 from Mexican *R. microplus*

H^a^	F^b^	Gc (%)^c^	Fip^d^	SAP’s^e^	Di (%)^f^
1	28	22.2	9/10	22/616	3.5
2	18	14.2	9/10	19/616	3.1
3	6	4.7	4/10	19/616	3.1
4	6	4.7	4/10	22/616	3.5
5	4	3.1	3/10	46/616	7.4
6	3	2.3	2/10	21/616	3.4
7	2	1.5	2/10	20/616	3.2
8	2	1.5	1/10	21/616	3.4
9	2	1.5	1/10	26/616	4.2
10–64	1	0.8	1/10	–	–

^a^Haplotype number. Each number (1–64) represents a haplotype. Numbers 1–9 represents the observed haplogroups

^b^Frequency of haplotype

^c^Genetic constitution

^d^Frequency of inter-population haplotype

^e^Single amino-acid polymorphism with respect to Yeerongpilly strain

^f^Percentage of divergence with respect to Yeerongpilly strain

### Correlation between genetic and geographic distances

A Mantel test was performed to find out the level of correlation existing between the matrices of genetic distances and geographic distances among all studied localities. The results of the test indicate that there is no correlation between genetic (*r* = 0.014) and geographical (*p* = 0.92) matrices distance. Accepting the null hypothesis, there is no distance isolation of the Bm86 gene, in the different sampled populations (*p* > 0.05).

**Fig. 2:**
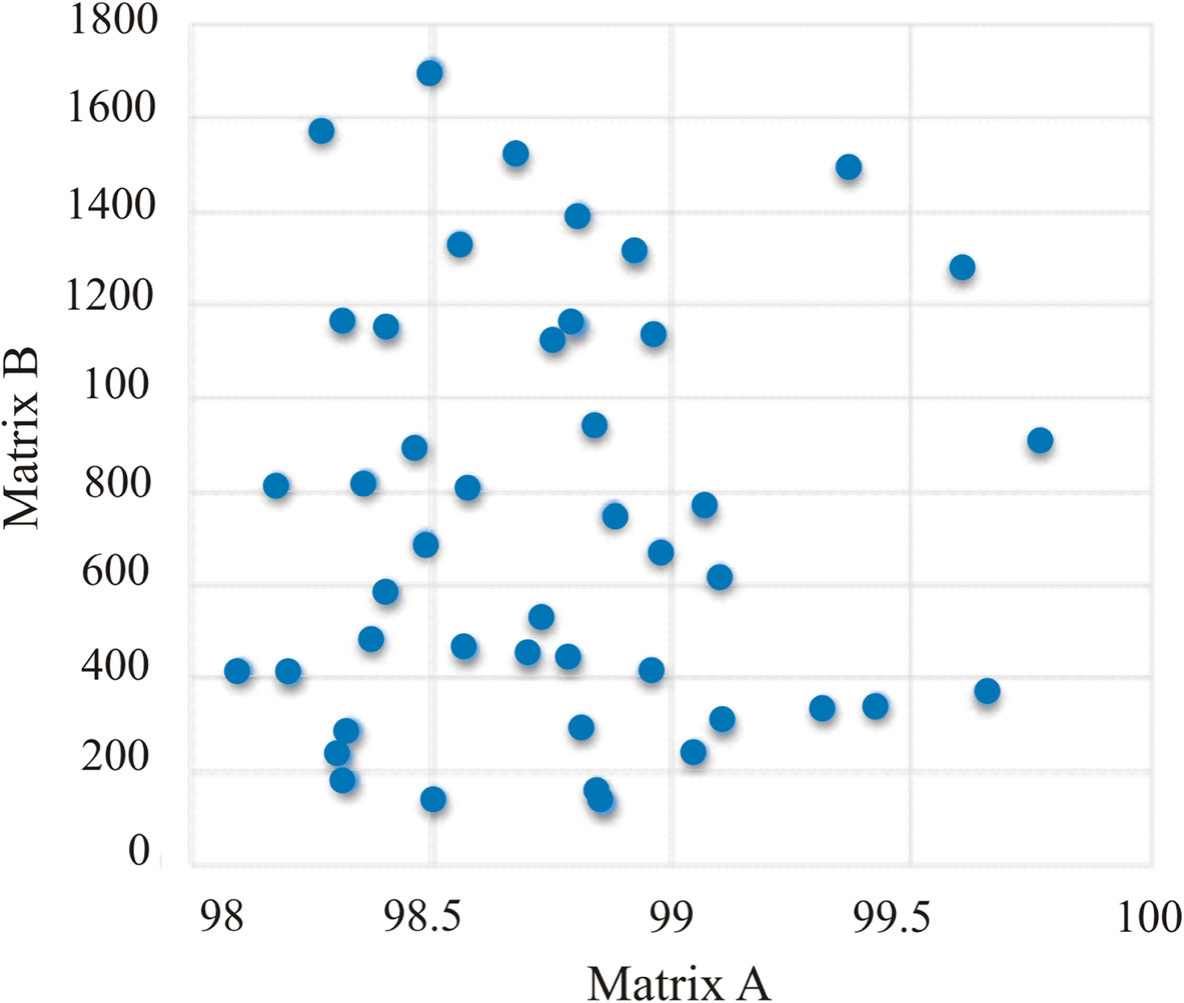
Mantel test, scatter plot showing correlation between genetic and geographic distances of the Bm86 haplotypes from ticks collected in different areas of the Mexican Republic. Real values for matrices are observed. **a**, gene distances in percentages of similarity; **b**, geographical distances in kilometers

A scatter plot (Fig. 2) was used to visually evaluate the correlation or cause-effect relationship for geographic and genetic distance matrices. The distribution of points in the diagram does not show a linear relationship between the variables.

The Pair-wise alignment in the identity matrix for amino acid sequences revealed that Texan sequences were similar to some of the Mexican ones. Comparison of Identity values shows identities ranging between 93 and 100% among Mexican and Texan sequences, while identities were 91–94% between sequences from Mexico and Thailand, 93–97% between sequences from Mexico and Bm86-CG from Brazil, and 86–88% between Mexican Bm86 and the Argentinian Bm95 sequences. Nucleotide sequences identity between Mexican and reference strain Yeerongpilly ranged from 92.6 to 96.9%.

### Minimum expansion network

**Fig. 3:**
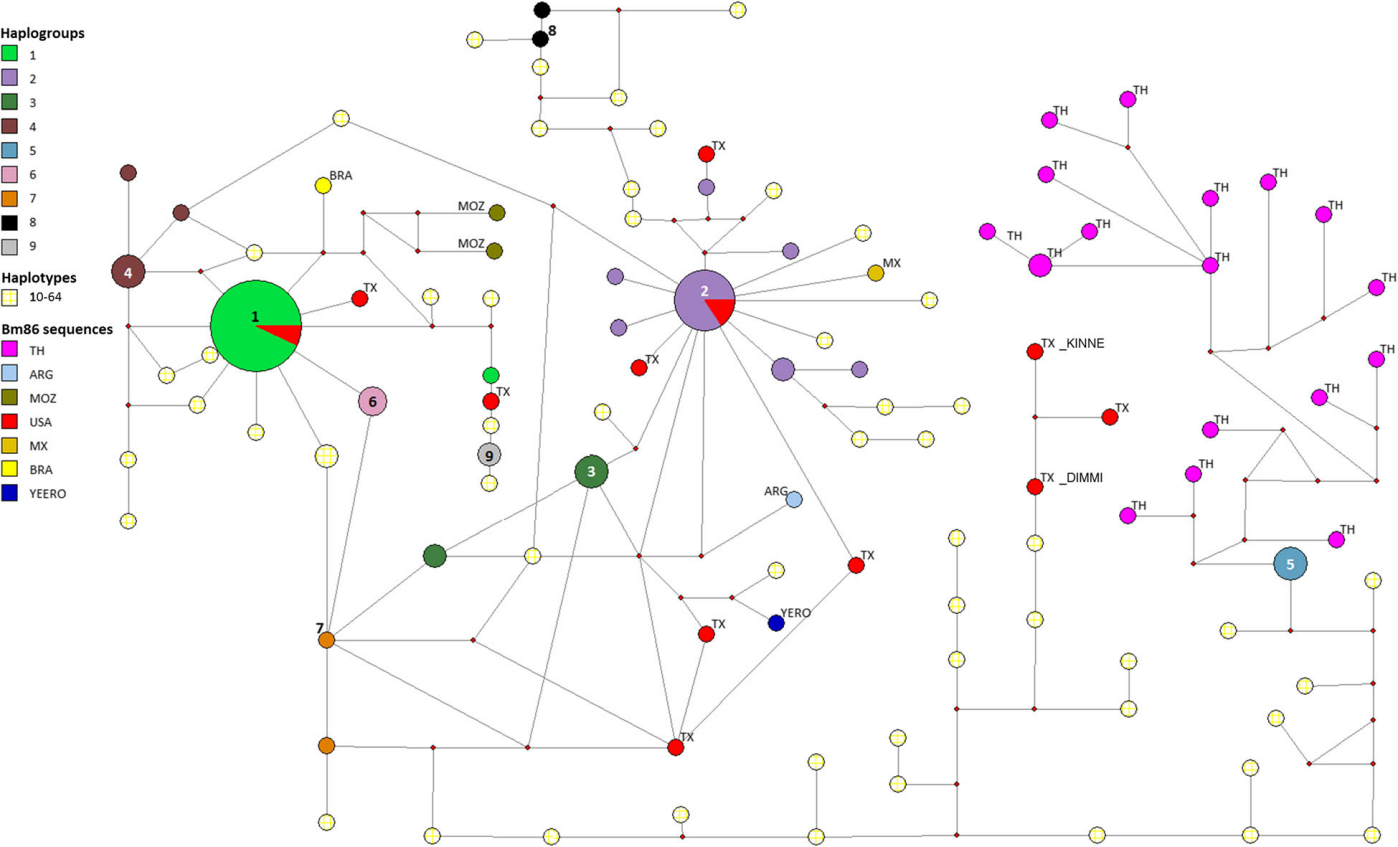
Minimum expansion network*.* It shows the frequency and relationship among haplotypes and haplogroups. Haplotypes and haplogroups are indicated with a colored label. Nine haplogroups can be observed. Haplogroups 1 and 2, containing the most abundant haplotypes. Haplotypes 10 to 64 are unique and have a random distribution in the network. GenBank Bm86 sequences included in the network come from Thailand (TH), Argentina (ARG), Mozambique (MOZ), United States of America (USA, TX), Mexico (MX), Brazil (BRA) and Australia (vaccine strain Yeerongpilly; YEERO)

The MEN diagram of the haplotypes does not show a defined phylogeographic structure. Figure 3 shows the phylogenetic structure of the 64 haplotypes found in Mexican isolates and those retrieved from GenBank. It should be noted that the size of the branches does not reflect the number of mutations of the derivatives. The diagram shows 9 haplogroups, however, haplogroups 1 and 2 are notable, not only for containing the most abundant haplotypes, but also because they show a pattern of dispersion within the localities sampled. Haplogroup 1 contains 28 sequences from Veracruz, Chiapas, Sinaloa, Morelos, Campeche, Colima, Zacatecas, Tabasco, and Nayarit. In comparison, haplogroup 2 contains 18 sequences from the same localities of haplogroup 1, although it excludes sequences from Chiapas and includes sequences from Guerrero. The rest of the haplotypes found in the present study are unique haplotypes.

Regarding the sequences found in GenBank, two of the Texan sequences are contained in haplogroup 1 and two more in haplogroup 2, the rest of the Texan sequences and Latin American reports: Argentina, Brazil, USA in addition to the only Mexican previous report, show a random distribution. In a more prolonged expansion, by putative nodes of divergence, it can be observed that the two sequences from Mozambique arise from haplogroup 1. Something similar occurs with the Yeerongpilly reference sequence, which can be included in haplogroup 2. Lastly, in a monophyletic cluster, we can see all the sequences of Thailand arising from haplogroup 5.

### Phylogenetic analysis

**Fig. 4:**
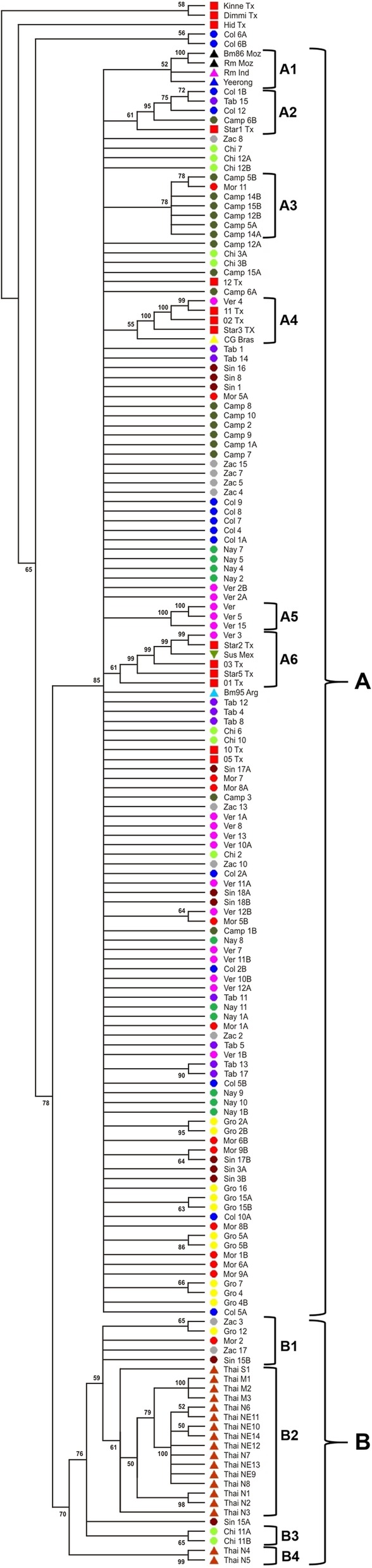
Molecular Phylogenetic analysis by Maximum Likelihood method. Tree reconstruction of evolutionary distances among putative sequences of the Bm86 gene of Mexican tick populations was inferred by using the Maximum Likelihood method based on the Kimura 2-parameter model. A discrete Gamma distribution was used to model evolutionary rate differences among sites (2 categories (+G, parameter = 0.0500)). Bootstrap evaluation adjusted for 1000 permutation repetitions. The main clades considered here are shown in brackets marked as A and B. Clade B has 5 subclades labeled from B1 to B5. A total of 165 complete sequences, considering amino acid residues from 20 to 627 from the Bm86 were used in the analysis, including 125 Mexican sequences (localities are marked with circles of different colors) and GenBank sequences: 14 from Texas (USA, orange triangles); 18 from Thailand (brown triangles); 1 from Australia (blue triangle); 1 from Argentina (light blue triangle); 1 from India (pink triangle); 1 from Brazil (yellow triangle); the only previous Mexican (green triangle) and 2 Mozambique sequences used as outgroup (black triangles)

The phylogenetic tree constructed with the Bm86 complete cDNA sequences found in the present report, plus the sequences available in GenBank, shows two main clades, **A** and **B**. Clade **A** includes the largest number of sequences, however, most of them do not form part of any identifiable subclade and have differences that make them unique. Clade **A** also contains six small subclades (A1-A6). Subclade A1 is formed by four sequences coming from the Australian, African and Asian continents: Australia (Yeerongpilly) (*n* = 1), Mozambique (*n* = 2) and India (n = 1). Subclade A2 corresponds to five sequences coming from Colima (n = 2), Tabasco (n = 1), Campeche (n = 1) and Texas (n = 1). To the subclade A3, belong seven sequences from Campeche (*n* = 6) and Morelos (n = 1). Subclade A4 is formed by five sequences corresponding to Texas (*n* = 3), Veracruz (n = 1) and Brazil (n = 1). Subclade A5 is formed by 3 sequences from the state of Veracruz, and subclade A6 is formed by six sequences coming from Veracruz (n = 1), Texas (*n* = 4) and Tamaulipas (Sus Mex sequence) (n = 1). Clade **B** is formed by 26 sequences that show more divergence than clade A. It forms four subclades: three small subclades (B1, B3 and B4) and a large one (B2). Subclade B1 is formed by 5 Mexican sequences coming from 4 different regions. Subclade B2 is mainly represented by sequences from Thailand (*n* = 16). Subclades B3 and B4 are formed by two sequences each from Chiapas or Thailand, respectively. Standing independently from clades A and B are; one sequence from Texas, two from Colima and the two *R. anulatus* (Kinne and Dimmi) sequences used for tree rooting (Fig. 4).

## Discussion

There are two commercial vaccines based on the recombinant antigen Bm86: Tick-GARD™ (Australian antigen Yeerongpilly) and GAVAC® (Cuban antigen Camcord, available in Mexico). However, the Camcord sequence is not available in GenBank and considering that these two protein sequences are identical except for a single amino acid [33], we considered the Yeerongpilly as reference sequence for all analyses and comparisons purposes in this work.

The lack of specificity of the commercial anti-tick vaccines, is probably due to the existence of genetic polymorphism among the *R. microplus* populations, as has already been reported [22, 25, 26, 33, 36, 37, 52–54]. In Mexico, it was previously suggested that no significant differences exist between Bm86 protein sequences from Mexican tick populations and the rBm86 protein derived from Yeerongpilly [26]. This is inaccurate. The analysis carried out in that work, that correlated genetic diversity and efficacy, was based in only two (partial) Bm86 protein sequences (35 amino acids, Tuxpan and Mora; GenBank accession numbers AF150892 and AF150893, respectively) [36]. Other than those two sequences, only one single Mexican complete cDNA Bm86 sequence (1827pb and 608 aa, GenBank:FJ456928) [55] had been reported at the time this study was conducted. Our findings regarding the genetic variability found within 10 Mexican populations sampled, challenge the previous knowledge on the influence of genetic polymorphism over the effectiveness of the commercial vaccines in Mexico.

Here, 125 complete cDNA sequences of the Bm86 gene obtained from tick specimens collected from 10 different regions of Mexico were analyzed. Not all samples analyzed by rtPCR produced amplicons. This might be partially explained by sample handling and processing and presence of PCR inhibitors. However, this could also be associated with primers specificity, particularly if one considers: 1) the polymorphism of sequence Bm86 observed in the present study, and 2) the presence of samples that yielded only 2 or 3 of the 4 amplicons that covered the expected Bm86 sequence, but that were not included in the study as they were not complete (data not shown). These RNA samples should be reanalyzed with new sets of primers to validate whether the lack of amplification was due to the large allele polymorphism found in the present report. It is important to note that the polymorphism of Bm86 cDNA sequences found in the present report is high, considering that about 50% of the haplotypes found are unique. However, this polymorphism is not extraordinarily high, as it is consistent with the polymorphism observed in similar studies developed in other regions of the world, such as those conducted in Texas and Thailand [22, 33].

The divergence of the commercial antigen compared against the sequences in this study was higher than 2.8%. This value has been estimated as the higher limit of differences in amino acid sequences that allows efficient vaccine performance [36]. This result also suggests that the low effectiveness of the commercial vaccine in Mexico might be explained by Bm86 polymorphism. Although we expected some divergence between the sequences from Mexican ticks and the one used in the commercial vaccine, it was somehow surprising not to find *R. microplus* specimens in Mexico with the same haplotype as the one from the commercial antigen. This could be probably explained by the fact that the commercial vaccine has been used for over 15 years in Mexico, so it is plausible that such negative selection could have eliminated a large amount of individuals with this genotype from the tick population. More extensive studies covering other regions of Mexico and with higher sample numbers should be carried out to test this hypothesis.

The Mantel test revealed no correlation between the genetic distances’ matrix of the Bm86 sequence and the geographical distances’ matrix of the studied regions, so the observed haplotypes are not confined to a region. It would be difficult to explain ticks’ Bm86 gene geographic-flow, if it is not associated with the geographic flow of cattle. Detailed databases on the flow of livestock across of national territory are not available on official sites for the open public. It could be that, actions such as: cattle import and/or export Mexico-USA, cattle translocation within the Mexican territory and layout of commercial routes and the difficulty associated with the tick control with pesticides, have played a very important role in the territorial distribution of the observed haplotypes. This data suggest that a vaccine design should include the epitopes for most representative haplogroups.

The minimum expansion network shows the frequency and the relation kept among haplotypes and haplogroups in a diagrammatic way. This diagram exhibits the existence of 9 defined haplogroups with Mexican sequences found in all of them. In this network, haplogroups 1, 4, 6 and 7 are closely related among them, haplogroups 2 and 3 are closely related between them, and haplogroups number 5, 8 and 9 remain somehow isolated. The distribution of the minimum expansion network suggests the occurrence of a significant expansion event for the Bm86 sequence, and such distribution suggests a high level of gene flow and Bm86 polymorphism for the analyzed locations. Since the DNA sequence phylogenetic tree and the minimum expansion network showed a random distribution of the haplotypes, it is not possible to define a phylogeographic pattern related to divergence. Haplogroups 1 and 2 are the most robust and are present in 9 out of 10 regions studied. The seven other haplogroups were formed from two and six individuals to the remaining 55 sequences form unique haplotypes. This information is relevant because it highlights the haplogroups sequences that should be prioritized for vaccine design. The divergence found for these 9 haplogroups ranged from 3.1 to 7.4%. This is an interesting finding because, as mentioned before, it has been shown that when the rBm86 vaccine antigen sequence show differences higher than 2.8% compared with the Bm86 target amino acid sequence, the vaccine will show a reduced effectiveness [22, 25, 29, 36, 56]. These data suggest that sequences from more than one haplogroup should be considered to design a vaccine that aims to be effective against most *R. microplus* tick populations (Table 3 and Fig. 3).

The Maximum Likelihood method used for the reconstruction of the DNA sequence phylogenetic tree shows two main clades: the America’s and Thailand’s. This data confirms previous observations by Kaewmongkol and coworkers [22], who compared Bm86 cDNA squences found in Thailand with some sequences reported from America. Most Mexican sequences remained evolutionary separate from Thailand’s clade. However, seven sequences from Mexico are evolutionary close to Thailand’s sequences. This sequence distribution could be attributed to commercialization of cattle that was not appropriately inspected for tick control. The seven Thailand-type genotypes found in Mexico, were collected from five distant states: Guerrero, Sinaloa, Morelos, Zacatecas and Chiapas, indicating the wide distribution of Thailand-type genotypes in Mexico. Considering that the sample size was not designed to detect particular genotypes, that sample size was not large enough to determine the real distribution of Thailand-type genotypes in Mexico, and that the dispersion of these haplotypes was observed in five distant regions; it can be hypothesized that this kind of genotypes should have a larger distribution than the one observed in the present report, and that an additional study should be conducted to find out a more precise distribution of Thailand-type genotypes in Mexico.

From the evolutionary point of view, the most closely related sequences to the Yeerongpilly Bm86 cDNA sequence from Australia (the vaccine genotype) are those reported for Mozambique [2] and India [1]. However, it is interesting to note that from the minimal expansion network, constructed with amino acid sequences, that the Yeerongpilly sequence appears somehow isolated. When comparing the phylogenetic tree with the minimal expansion network, it can be observed that America and Thailand are grouped as separate clusters. This information suggests that al least two different vaccines should be developed to target these two phenotypic tick clades. The Bm95 sequence, derived from an Argentinian tick has also been tested as vaccine in South America, seems also isolated in the MEN. For practical printing reasons the phylogenetic tree was edited to have equal size branches, because the original tree format was too wide to be placed in a printable page. But in that tree, the Bm95 sequence also seemed to diverge considerably from most sequences analyzed in the present report, and it doesn’t seem to be the right option to develop an anti-tick vaccine for the genotypes circulating in North America or Thailand. Data analysis suggests that more than one genotype should be used for the design of one or more vaccines to target the wide number of genotypes present in *R. microplus* populations.

The relationship between some Texan and Mexican Bm86 sequences, and the lack of geographic delimitation of the majority of the Mexican sequences could be explained by the movement of tick-infested livestock, both within the Mexican territory and across the American border. The latter is supported by data from Skaggs et al. (2004), showing Texas as one of the largest importer of Mexican cattle into the United States. Cattle commerce and mobility is regulated by Mexican and American federal laws, and both parties are required to inspect cattle for ticks and tick-borne diseases, such as babesiosis and anaplasmosis. Despite the official inspection protocols followed by both countries to prevent tick dissemination, ticks with similar Bm86 sequences are found in Texas and different states of Mexico. Epidemiologically, the tick issue at the border between Mexico and the United States might not be limited to cattle commerce, since *R. microplus* is also found in wild Artiodactyla, such as deer and antelopes, and these animals are free to move back and forth between both countries [57]. In this scenario, the ticks could be dispersed by wild animals throughout the Mexican and US territories border, despite cattle inspection performed by the tick control programs from both countries. This cannot be evaluated at the moment, as there are no extensive databases recording the flow and interaction of livestock and wild animals in Mexico.

## Conclusion

The high level of polymorphism of Bm86 sequences found in populations from 10 different states of Mexico and their differences from the sequences used to produce the commercial vaccine currently administered to Mexican cattle, highlights the need to develop new anti-tick immunization agents. Future studies should aim to increase the current database of Mexican Bm86 sequences. Sampling sites should include different areas from the states considered for this study, as well as from other states that were not part of this work. Additionally, studies aiming to detect antigenic regions and epitopes through computational analysis should be conducted to design appropriate antigens to successfully develop a new, more effective, wide-spectrum anti-tick vaccine.

## Data Availability

DNA and amino acid sequences reported in this paper were deposited in the GenBank repository, however sequence data and accession numbers for submitted sequences are not publicly available until the year 2021, because this information will be probably used to produce a commercial vaccine and should be kept temporarily confidential, until patent it is registered.
